# Recent Advances of Macromolecular Hydrogels for Enzyme Immobilization in the Food Products

**DOI:** 10.34172/apb.2022.043

**Published:** 2021-07-04

**Authors:** Leila Yavari Maroufi, Mohsen Rashidi, Mahnaz Tabibiazar, Maryam Mohammadi, Akram Pezeshki, Marjan Ghorbani

**Affiliations:** ^1^Department of Food Science and Technology, Faculty of Nutrition and Food Science, Tabriz University of Medical Sciences, Tabriz, Iran.; ^2^Department of Pharmacology, Faculty of Medicine, Mazandaran University of Medical Sciences, Sari, Iran.; ^3^Department of Food Science and Technology, Faculty of Agriculture, University of Tabriz, Tabriz, Iran.; ^4^Nutrition Research Center, Tabriz University of Medical Sciences, Tabriz, Iran.

**Keywords:** Hydrogels, Enzyme immobilization, Food industry

## Abstract

Enzymes are one of the main biocatalysts with various applications in the food industry. Stabilization of enzymes on insoluble carriers is important due to the low reuse, low operational stability, and high cost in applications. The immobility and the type of carrier affect the activity of the immobile enzyme. Hydrogels are three-dimensionally cross-linked macromolecular network structures designed from various polymers. Hydrogels can provide a matrix for an immobile enzyme due to their extraordinary properties such as high water absorbing capacity, carrier of bioactive substances and enzymes, biocompatibility, safety, and biodegradability. Therefore, this study mainly focuses on some enzymes (lactase, lipases, amylases, pectinase, protease, glucose oxidase) that are of special importance in the food industry. These enzymes could be immobilized in the hydrogels constructed of macromolecules such as kappa-carrageenan, chitosan, Arabic gum, pectin, alginate, and cellulose. At last, in the preparation of these hydrogels, different enzyme immobilization methods in macromolecular hydrogels, and effect of hydrogels on enzyme activity were discussed.

## Introduction

**Figure 1:**
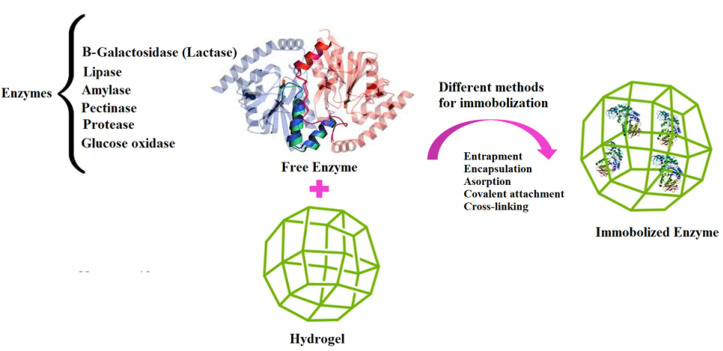
The scheme summarized the different enzymes which can be immobilized on hydrogels with different methods.

Enzymes are a type of biocatalysts, widely applied in several applications in the food industry, such as baking, beverages, meat, dairy, fats and oils, as an effective, safe and eco-friendly alternative for food production. Enzymes have been used as food preservatives for long years, and nowadays they are enabling a variety of food industries to give the quality and stability of their products, along with better production efficiency. They provide clean, environment friendly and specific methods for biochemical reactions in moderate conditions.^1-3^ However, the use of enzymes is limited due to their high cost and low reusability. Moreover, the lack of proper mechanism to protect enzymes against protease attack, occurring in almost all biological systems, is another major hurdle to achieve optimal activity.^4^ Additionally, the low operational stability of some enzymes during any biochemical reaction is problematic. Therefore, enzyme stabilization is the main objective of enzyme technology. The attainment of stable and active enzymes is a highly challenging effort. In order to overcome these limitations, the immobilization of enzymes with functional efficiency is useful to solve the enzyme problems and decrease the costs. The immobilization method involves the inclusion of enzymes in matrices or binding them on various surfaces.^5,6^ The immobilization of enzymes on hydrophobic supports is a general method. There are various chemical catalyst carriers to immobilize enzymes, one of which is the use of hydrogel matrixes, hydrogels may be used as appropriate carriers for enzymes.^3^ The ideal carrier matrix should have the following properties: (a) to be economical, (b) inertness, (c) stability, (d) physical strength, (e) ability to enhance enzyme specificity/activity, (f) regenerability, (g) ability to reduce product inhibition, and (h) ability to prevent nonspecific adsorption and bacterial contamination. Immobilization usually stabilizes the enzyme structure, allowing the hydrogels’ use under harsh environmental conditions (pH, temperature, and presence of organic solvents).^7^ Hydrogels are water-insoluble three-dimensional hydrophilic polymer networks that possess all the mentioned ideal carrier properties with a high ability to retain water and other liquids.^8^ Therefore, the aquatic environment of hydrogels can reduce the denaturation of enzymes and help their catalytic function.^9,10^ Hydrogels, as smart materials, can respond to many environmental stimuli, including temperature,^11^ pH^12^ by showing changes in structure, shape and interaction with their loaded substrates. They have several applications, such as drug delivery, release,^13^ enzyme trapping, releasing^8^ and biosensor.^14^ In this study, some important enzymes in the food industry immobilized in hydrogels, various natural polymers used in the preparation of hydrogels, methods of enzyme immobilization in matrix hydrogels and hydrogels effect on the activity of enzymes are discussed. Figure 1 summarized the different enzymes which can be immobilized on hydrogels with different methods.

## Enzymes

The enzymes play a variety of roles in the food industry. Some of these roles are listed in Table 1.

**Table 1:** Application of immobilized enzymes in food industry

**Enzyme**	**Application in food industry**	**REF**
β-Galactosidase (lactase)	β-Galactosidase is an enzyme widely used in dairy products to hydrolyze of lactose, makes it possible consumption of dairy products for people who are lactose intolerance, used to produce oligosaccharides, that are known as prebiotic products	^15-18^
Lipase	play an important role in the dairy industry, including hydrolyze milk fat, accelerating cheese ripen, increase the flavor of cheese and lipolysis of butter. In lipid industry, lipases can be applied to retailoring of animal and vegetable oils	^19-21^
Amylase	Amylase is most important enzymes in the bread industry, which breaks down damaged starch in wheat flour into small dextrins and strengthens the dough, resulting in improved bread volume	^22-24^
Pectinase	extracting and clarifying wine and fruit juices, fruit maceration, reducing the viscosity of fruit juices, extraction of vegetable oil, coffee and tea fermentation and valorization of industrial wastes	^25-27^
Protease	production of cheese by coagulating milk, improving the digestibility and nutritional value of biscuits, pastries, wafers, cookies through protein hydrolysis, the production of gluten-free pasta and the production of functional products	^28-30^
Glucose oxidase	oxygen scavenger, catalyzes the reaction of glucose and oxygen and remove oxygen from food and beverages to prolong their shelf life	^31,32^

### β-Galactosidase (lactase)

β-Galactosidase is from the hydrolase family of enzymes. It is an enzyme usually used to hydrolyze lactose in dairy products. Lactose is the predominant disaccharide in milk and dairy products that some people are unable to consume due to sensitivity. The β-galactosidase enzyme by lactose hydrolysis, makes the consumption of dairy products possible for people with lactose intolerance. Also recently, this enzyme has been used to produce oligosaccharides, known as prebiotic products. Therefore, the use of the β-galactosidase enzyme facilitates the production of useful products in the food industry. Since enzymes have low stability, their immobilization and stabilization on suitable carriers are essential. Immobilization of enzymes is an easy procedure with several benefits, including enzyme reusability, persistent process, increased stability under operation and storage state.^15-18^

### Lipases

One of the widely used biocatalysts is lipase. Lipases are from the hydrolase family of enzymes. They are effective enzymes with various applications in medicine, pharmaceuticals, cosmetics, detergents, paper production and the food industry. They are good catalysts for the production of food additives and ingredients. They have great potential for synthesizing short-chain esters to be used in the food industry as flavor modifiers or fragrance compositions. Lipases play an important role in the dairy industry, including hydrolyzing milk fat, accelerating cheese ripen, increasing the flavor of cheese and lipolyzing butter. In the lipid industry, lipases can be applied to retailor animal and vegetable oils. The industrial use of lipases is limited due to the high cost of their production, the lack of long-term stability and difficulty in recycling them; thus, immobilizing them on suitable matrices, such as hydrogels, can be very effective.^19-21^

### Amylases

Amylases are from the hydrolase family of enzymes. They are widely found in microbial, plant, and animal sources and are one of the important industrial enzymes with many applications in the food and beverage industries. Amylase is the essential enzyme in the bread industry, which breaks down damaged starch in wheat flour into small dextrins and strengthens the dough, resulting in improved bread volume. Further, small oligosaccharides and sugars such as glucose and maltose, produced by this enzyme increase Millard response responsible for browning the shell and creating an attractive cooked taste.^22-24^

### Pectinase

Pectinase is from the hydrolase family of enzymes. The enzyme is used in processing pectin, the main component in the middle lamella of the plant cell wall. Pectinases are widely used in the food industry, such as extracting and clarifying wine and fruit juices, macerating fruit, reducing the viscosity of fruit juices, extracting vegetable oil, fermenting coffee and tea, and valorizing industrial wastes; due to these extensive applications, they make up 25% of the world’s enzymes. Though, like many other industrial enzymes, pectinase has a limited yield and low efficiency in its economic generation.^25-27^

### Protease

The protease enzyme belongs to the family of hydrolases. The origin of protease enzymes is plant, animal, and microbial. Protease or peptidase is an enzyme that hydrolyzes peptide bonds, which is the main commercial and industrial enzyme. The proteases represent the largest group of commercially available enzymes worldwide, accounting for 60% of the industrial enzymes market due to their wide range of applications in food, beverage, detergent, medical diagnosis, leather industries, as well as research and development activities. In the food industry, it is widely used in producing cheese by coagulating milk, improving the digestibility and nutritional value of biscuits, pastries, wafers, cookies through protein hydrolysis, producing gluten-free pasta and producing functional products. Therefore, its immobilization in the hydrogel matrix reduces costs and makes it easy to be widely used in the food industry.^28-30^

### Glucose oxidase

Glucose oxidase is an oxidoreductase that catalyzes the oxidation of glucose to gluconic acid and hydrogen peroxide. It has many uses; for example, it scavenges oxygen in the food industry effectively, catalyzes the reaction of glucose and oxygen that generates glucuronic acid, and successfully removes oxygen from food and beverages to prolong their shelf life.^31,32^

## Hydrogel matrixes as enzyme carriers

Hydrogels are three-dimensional, polymeric and hydrophilic networks. They are formed from both synthetic and natural hydrophilic polymers that are water-insoluble, able to swell, absorb, and retain major amounts of water. Over the years, researchers have defined hydrogels in many different ways. The most commonly used definition is that the hydrogel is a water-swollen and cross-linked polymeric network, produced by the simple reaction of one or more monomer/polymer/cross-linker units. One more description is that it is a polymeric material that exhibits the ability to swell and retain a large amount of water in its three-dimensional network, however, will not dissolve in water.^33,34^ Hydrogels have good biocompatibility and can provide a suitable microenvironment^32^ and they are widely used in different fields including drug delivery systems, tissue engineering, protein and cell immobilization, agriculture and horticulture and food industry.^35,36^ In recent years, development of responsive hydrogels has been observed in various field. In particular, hydrogels of polymers such as chitosan, alginate, kappa-carrageenan, etc. have been used as supports for enzyme immobilization. Some studies have reported the immobilization of various enzymes including lipase, lactase, protease, and amylase on polymer-based hydrogels. Enzymes immobilization on soft and solid supports, such as hydrogels, is an efficient procedure amongst diverse enzyme immobilization techniques. Because of retaining a large amount of water inside the three-dimensional network, they provide efficient physiological conditions for enzyme activity. The aqueous environment of polymeric hydrogels can reduce the denaturation of enzymes and help enzymatic functions. Therefore, it can be expected to maintain enzyme activity due to the immobility in the hydrogel polymer matrix.^37^

### Kappa-carrageenan based hydrogel

Kappa-carrageenan, thermo-reversible gel, is a linear, negatively charged sulfated polysaccharide extracted from marine red algae. Kappa-carrageenan is widely used in food, cosmetics, and drug controlled release and encapsulation due to high biodegradability and biocompatibility. In the food field, due to gelling capabilities in the presence of counter-ions, especially K, it is used for many applications.^38-40^ Zhang et al^40^ immobilized β-galactosidase enzymes into kappa-carrageenan-based hydrogel beads. As shown in studies conducted by them, the immobilization of β-galactosidase enzyme into carrageenan-based bead hydrogels improved enzyme activity at pH and medium temperature conditions; the physicochemical origin of this effect was attributed to the ability of K ions used to cross-link the polysaccharide chains to increase the stability and activity of β-galactosidase.

### Chitosan based hydrogel

Chitosan-based hydrogels have received substantial interest recently in enzyme immobilization, drug delivery, agriculture, biomedicine and food industry. Chitosan is a nontoxic natural polymer produced by the deacetylation of chitin and compound of glucosamine (70%) and acetylglucosamine (30%) units with a molecular weight ranging from 50 to 1000 kDa. It is the second most abundant polysaccharide in nature after cellulose.^41^ Chitosan due to beneficial hydrophilic, cationic, and biodegradable properties, applied in several fields, such as agricultural, food, and pharmaceutical industries.^42^ This natural polymer has a high potential to produce gels, films, fibers and particularly hydrogels.^43–46^ Facin et al,^47^ Wolf et al,^48^ and Wolf and Paulino,^49^ and Ricardi et al^50^ immobilized β-galactosidase enzyme. Pereira et al^51^ immobilized lipase enzyme in the chitosan-based hydrogel and showed that chitosan-based hydrogels can be useful for carrying the enzymes.

### Arabic gum based hydrogel

Acacia gum, also known as Arabic gum, is an edible gum extracted from the trunks and branches of *Acacia senegal*and *Acacia seyal*rich in low-viscosity soluble fibers. A type of natural amorphous, non-toxic, water-soluble, odorless, colorless. and tasteless polysaccharide; it has been widely applied from ancient times to the present for different purposes in pharmaceutical, food, and other industries.^52,53^ Its molecular structure has a complex mixture of glycoproteins and sugars acting as active sites on immobilization processes. It includes mainly of polysaccharides arabinose and galactose, calcium, magnesium, and potassium salts.^54^ Wolf et al,^48^ and Wolf and Paulino ^49^ immobilized β-galactosidase enzymes in the Arabic gum-based hydrogel and showed Arabic gum-based hydrogels to be good solid matrices for the β-galactosidase enzyme immobilization, able to be used for hydrolysis of lactose in dairy foods.

### Pectin based hydrogel

Pectin is a frequently used thickening and gelling agent in several food and non-food industries with high consumer acceptance. It is a natural heteropolysaccharide extracted from the skin of apple and citrus fruits. Pectin can be applied in various food applications, being a gelling agent, emulsifier, stabilizer, glazing agent, and fat replacer.^55^ Predominantly, it consists of α-1,4-linked galacturonic acid-based units. Pectin due to its unique properties including biocompatibility, degradability, and great transparency, can be used as a matrix to carry useful materials such as enzymes.^34,56^ Cargnin et al immobilized β-Galactosidase enzyme in the pectin-based hydrogel and indicated it to be excellent solid supports for the immobilization of enzymes. The immobilization of β-Galactosidase in pectin-based hydrogels can be used in the hydrolysis of lactose of dairy products for lactose-intolerant individuals^18^; also, Hasanah et al^57^ immobilized lipase enzyme in the pectin-based hydrogel.

### Alginate based hydrogel

Alginate is a natural polymer. It, due to its properties such as non-toxicity, biocompatibility, low cost, gelation, chemically compatibility, availability, and degradability, is a suitable polymer for many scientific studies.^58^ In the food industry, it is a favorite ingredient, food additive, and carrier of effective ingredients in alginate gel encapsulation. Alginate, as an anionic polysaccharide, can be modified using chemical and physical reactions to be a good candidate for three-dimensional (3D) scaffolding derivatives such as hydrogels, microspheres, microcapsules, sponges, foams, and fibers.^59-61^ Fabra et al^62^ and Traffano-Schiffo et al^63^ immobilized β-Galactosidase enzyme; Oliveira et al^64^ immobilized pectinase enzyme; also, Mohammadi et al^65^ immobilized lipase enzyme in the alginate-based hydrogel; they showed that in order to better maintain the activity of the enzyme in the alginate matrix, alginate alone was not enough and the

### Cellulose based hydrogel

Cellulose is the most abundant polymer in nature, it is found in natural plants and fibers including cotton and linen. Cellulose is the starting material for a wide range of uses in the food industry as food additives and gelling agents.^66^ Cellulose based hydrogels are important due to their biocompatibility, non-toxicity and natural originality; they have potential to be used in dye or metal ion adsorption, drug delivery, and enzyme support.^67-69^ Park et al^70^ and Jo et al^71^ immobilized lipase enzyme in cellulose-based hydrogel and showed that cellulose hydrogel could be applied as a support for lipase and suitable for the immobilization of enzymes.

### Polyacrylamide based hydrogel

Polyacrylamide (PAAm), including acrylamide (AM), is a type of synthetic polymers that have several advantages, such as good flexibility, biocompatibility, and high solubility in water. It is used widely in liquid–solid separation in water and waste treatment, paper making, processing of minerals in mining, and oil recovery enhancement. Cross-linked polyacrylamide is used in the food industry as coating, films, and gelling agents. PAAm is a greatly utilized synthetic polymers in hydrogel production due to its great hydrophilicity. It also can be applied to immobilization of enzymes^72–74^; for example, Mulko et al^22^ successfully immobilized alpha amylase enzyme in the PAAm-based hydrogel.

### Polyvinyl alcohol (PVA) based hydrogel

PVA is a non-toxic, soluble (in water), semi-crystalline plastic, synthetic, and biocompatible polymer. It is a linear synthetic polymer produced by polyvinyl acetate hydrolysis. Due to its great properties, such as solvent resistance, mechanical efficiency, water high solubility. and eco-friendly, it is widely used in the preparation of hydrogels.^75,76^ The internal network of polyvinyl alcohol hydrogel has free water, crystalline and swollen amorphous PVA domains; it creates a porous structure and can be effective for various applications, including enzyme immobilization.^77,78^ As shown in Table 2, the various polymer based hydrogels was used in this field.

**Table 2:** Different types of hydrogels for immobilization of enzymes.

**Group of polymers of hydrogel**	**Hydrogel base**	**Enzyme**	**Ref**
Biopolymers	Kappa-carrageenan	β-Galactosidase	^38–40^
	Chitosan	β-Galactosidase	^47–49^
		Lipase	^51^
	Arabic gum	β-Galactosidase	^48,49^
	Pectin	β-Galactosidase	^18^
		Lipase	^57^
	Alginate	β-Galactosidase	^62,63^
		Lipase	^65^
		Pectinase	^64^
	Cellulose	Lipase	^70,71^
Synthetic polymers	Polyacrylamide	Alpha amylase	^22^
	Polyvinyl alcohol (PVA)	Protease	^77^
		Glucose oxidase	^78^

## Enzyme immobilization method in hydrogel

**Figure 2:**
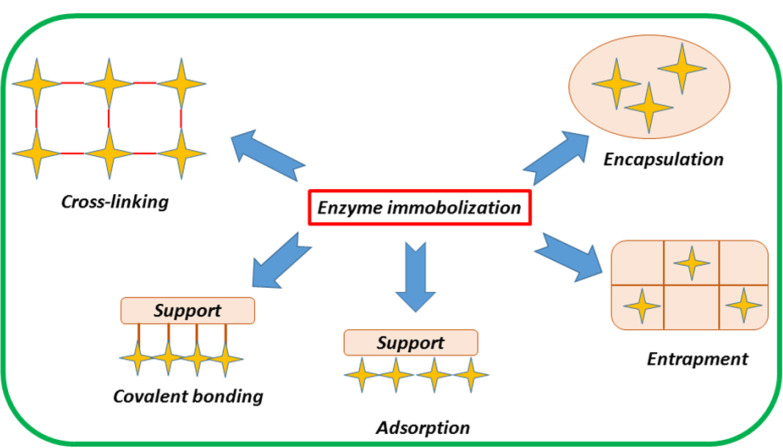
Different enzyme immobilization methods.

There are different methods to immobilize enzymes. As can realize from Figure 2, these methods are generally divided into two types: physical and chemical. In the former, there is a weak interaction among the enzyme and the carrier substance, while in the latter, there is a strong interaction due to the presence of covalent bonds. These immobilization methods are very important since the stability and long-term use of the enzyme depend on them. Common methods of enzyme immobilization include adsorption, encapsulation, entrapment, covalent attachment, and cross-linking.^79-81^ In addition, each of these methods has advantages and disadvantages that are briefly listed in Table 3.

**Table 3:** The advantages and disadvantages of different enzyme immobilization techniques

**Technique**	**Advantages**	**disadvantages**
Encapsulation/ Entrapment	Protection of enzyme activity, persistent action	Limitation of enzyme loading, catalysis carried out at interphase enzyme/substrate, mass transfer limitations
Adsorption	Easy and cheap, without the need to use reagents, great catalytic activity	Low stability, poor bonding on supports
Covalent attachment	Powerful bonding, inhibition of enzyme leakage, high thermal stability, increased operational stability, compatible with special process	Limitation of enzyme mobility, reduce of enzyme activity, conformational restriction
Cross-linking	Powerful bonding, prevention of leakage, reduce of desorption, easy to reuse	Loss of the enzyme activity, reduce of diffusion rate, weak mechanical properties, limitation of mass transfer

### Entrapment and encapsulation

The caging of enzyme can be achieved by any of the following strategies: (1) by inclusion if enzyme within a highly cross-linked polymer matrix, (2) by enzyme dissolution in a nonaqueous phase, or (3) by separating enzyme from a bulk solution by using a semipermeable microcapsule. In this method the enzyme is not bound to the support matrix unlike other methods. When an enzyme is trapped inside a matrix, it is said to be encapsulated. Encapsulation is a physical method with advantages such as being inexpensive and easy; however, its most imperative benefit for enclosing is that no chemical change of the enzyme is required, not causing significant changes in the structure and activity of the enzyme. For this method, there are porous and gel-like matrices. Hydrogels, with their hydrophilic and very porous polymer network, can be the most suitable structure for this method that is more efficient than free enzyme. Enzymes are physically encapsulated in the hydrogel network during the sol-gel transition that is a comparatively mild process, tending to protect the structural integrity and activity of the enzymes. The only drawback that has been mentioned in these studies is the leakage of the enzyme out during storage in aqueous solutions^8,82^; in recent studies on immobilization of enzymes in hydrogel, the encapsulation method has been used.

There are various methods of enzymes entrapment like fiber entrapping, gel entrapping, microencapsulation, etc. In C. rugosa, when the lipase enzyme was entrapped in chitosan hydrogel, it showed enhanced enzyme activity and entrapment efficiency. It also prevented friability and leaching. This is mainly because the support matrix is biocompatible and nontoxic; receptive to chemical modifications because of its hydrophilic nature it has high affinity toward proteins.^83^

### Adsorption

In this method, the enzyme molecules adhere to the surface of the carrier matrix by a combination of hydrophobic interactions and the formation of various salt linkages per molecule of enzyme. Adsorption immobilization is a physical method that results from van der Waals and other noncovalent interactions, including hydrophobic interactions and hydrogen bonding electrostatic linkages among the support and the attached enzyme.^76^ Adsorption immobilization method is a naive, inexpensive, and reversible technique of enzyme immobilization. The adsorbed enzymes are usually resistant to proteolysis and aggregation because of their hydrophobic interaction with interfaces.^83^ Other benefits of this technique are: it supports the lowest activation, or no preactivation at all is required so that no reagent is needed; it shields against aggregation, proteolysis, and main interactions, which could disrupt enzyme and carrier potentials, and no working enzymes can be supplanted with new ones. The drawback of this technique is that the binding or linking forces among the enzyme and the carrier are weak from being established via hydrogen bonding, hydrophobic interactions, and ionic and van der Waals bonding forces.

It was reported that when Yarrowia lipolytica lipase was immobilized on octyl-agarose and octadecyl-sepa hydrogel beads supports by physical adsorption, resulted in greater stability, higher yields, better process control, and quite economical as compared to free lipase. This was mainly because of the hydrophobicity of octadecyl-sepa beads that increases the enzyme and support affinity.^83^

### Covalent attachment and cross-linking

Other technique is covalent attachment and cross-linking in which covalent bonds, in general, are generated due to chemical reactions between enzymes and supported materials. This method is mainly depending on the formation of covalent bond between the enzyme and the support material. Covalent bond formation between the enzyme and the matrix happens through the side chain amino acids like histidine, arginine, aspartic acid, etc. Covalent bonds can prevent enzyme leakage and improve the stability and reusability of enzymes; however, there is a high risk of enzyme denaturation, possibly modifying enzymes chemically. Covalent bond formation between the enzyme and the matrix happens through the side chain amino acids like histidine, arginine, aspartic acid, etc. However, the reactivity depends on the presence of different functional groups such as carboxyl group, amino group, indole group, phenolic group, sulfhydryl group, thiol group, imidazole group, and hydroxyl group. It requires, however, only low amounts of enzymes to be immobilized, and enzyme catalytic activity may be lost to some extent^84,85^; for instance, Pereira et al^51^ used covalent attachment method for immobilizing lipase in chitosan-based hydrogel and showed this method to be performed by adding glutaraldehyde and binding between free aldehyde groups and amine groups (NH_2_) lipase, performing better than physical methods. Maintenance of immobilized enzymes structural and functional properties is very important which can be played by a cross-linking agent. Glutaraldehyde is one such cross-linking agent, due to its solubility in aqueous solvents and can form stable inter- and intrasubunit covalent bonds, popularly used as functional cross-linker.

## Enzyme activity and release

Enzymes are applied as biocatalysts in the food industry. They are applied due to their different properties, such as selectivity, non-toxicity, usage of mild reaction conditions, and lack of secondary reactions. However, their use is limited due to low operational stability, low storage stability, and non-reusability. Therefore, the development of stable and recyclable enzymes for industrial applications is significant research effort.^86,87^ Polymeric hydrogels have recently emerged as a new matrix to immobilize enzymes, which can improve enzymatic activity and stability, and make them possible to be reused, and reduce costs. Hydrogels, due to their porous structure and water absorption properties, create a suitable environment for enzymatic activity and reduce enzymatic denaturation.^88^ The studies in this field clearly show that the immobilization of enzymes in hydrogels improves and even increases enzymatic activity as compared to the free state. For example, the encapsulation of lactase enzyme in carrageenan, chitosan, alginate, and pectin-based hydrogels have increased enzymatic activity and stability in different temperature and pH conditions.^18,89-91^ In a study conducted by Almulaiky et al the retention of alpha-amylase using PAAm-based hydrogels reached 97.5%, indicating the ability of this hydrogel to protect enzymes, making them reusable.^92^ Also, in a study conducted by de Rajdeo et al the immobilization of pectinase in alginate-based hydrogel showed high operational stability and maintained more than 80% of its initial activity after the third cycle of reuse.^93^

## Conclusion

Hydrogels are extensively applied in the food industry since they consist of safe and degradable hydrophilic polymers. In recent years, significant progress in design of enzyme immobilization, support matrix with different pore size, and surface modifications are developed. Designing ideal support material by modifying specific structural features required for a target enzyme is now possible by new simulations. The current review has provided a universal overview of the potentials of hydrogels for immobilizing enzymes to be applied in the food industry. β-Galactosidase, lipase, pectinase, amylase, protease, and glucose oxidase enzymes are widely applied in the food industry, and their use is limited due to the low stability and high cost. Hydrogels provide a suitable environment for enzyme activity and reduce enzyme denaturation due to their water absorption properties. Therefore, the immobilization of enzymes in polymeric hydrogels is a very effective approach in using them, leading to the optimal use of enzymes and cost reduction. It is our view that the future holds significant promise with increased usage of immobilized enzymes in pharmacological, clinical, food, biotechnological, and other industrial fields. Moreover, as the structure of enzyme and the mechanism of action is known, controlled immobilization methods can be developed in future.

## Ethical Issues

Not applicable.

## Conflict of Interest

The author declares no conflict of interest.
